# Supplementary material for: A clinical study to evaluate the safe and effective use of a new, single use stethoscope cover to enable reduction in pathogen transmission during auscultation
Source: Front Med (Lausanne). 2023 Jun 22;10:1179145. doi: 10.3389/fmed.2023.1179145 (PMC10324409; doi:10.3389/fmed.2023.1179145)
Supplement: Supplementary file 1 [file Data_Sheet_1.docx]

**SUPPLEMENTARY FILE****A clinical study to evaluate the safe and effective use of a new, a single use stethoscope cover to enable reduction in pathogen transmission during auscultation**

Timo Z. Nazari-Shafti^1,2^, Heike Meyborg^1,2^, Jasper Iske^1,2^, Maximilian J. Schloss^1^, Fabian Seeber^1^, Aljona Friedrich^1^, Vasileios Exarchos^1,2^, Anja Richter^1,2^, Volkmar Falk^1,2^, Maximilian Y Emmert^1,2^

**Author affiliations**

1. Deutsches Herzzentrum der Charité (DHZC), Department of Cardiothoracic and Vascular Surgery, Berlin, Germany
2. Charité – Universitätsmedizin Berlin, corporate member of Freie Universität Berlin and Humboldt-Universität zu Berlin, and Berlin Institute of Health, Berlin, Germany

**Corresponding author:**

Maximilian Y Emmert, MD, PhD

German Heart Center Berlin

Department of Cardiothoracic and Vascular Surgery

Augustenburger Platz 1

13353 Berlin, Germany

Tel.: +49 (30) 4593 2030

emmert@dhzb.de

**Study Registration**: EUDAMED no. CIV-21-09-037762

**LIST OF CONTENT**

**I. SUPPLEMENTARY METHODS**

1. **Inclusion Criteria**
2. **Exclusion Criteria**

**II. Supplementary Tables:**

Table S1: Numeric rating of the acoustic quality of auscultations with Stethoglove® stratified by gender

Table S2: Acoustic quality of auscultations (numeric) with Stethoglove^®^ stratified by BMI

Table S3: Acoustic quality of auscultations (numeric) with Stethoglove^®^ stratified by body site

1. **SUPPLEMENTARY METHODS**

### Inclusion Criteria

To participate in this study, the subjects had to comply with all of the following inclusion criteria:

- Adult male and female patients (≥18 years) elected for cardiac surgery
- Ability to understand the purpose and risks of the clinical study
- Signed and dated informed consent

### Exclusion Criteria

The subject was excluded from participation in the study if any of the following exclusion criteria applied:

- Known allergy or contact sensitization against the Stethoglove^®^ material (thermoplastic urethane, TPU)
- Patients with known HIV, Hepatitis B or C infections
- Legal incapacity or limited legal capacity
- Participation in an interventional clinical trial within 30 days prior to enrolment
- Employees of the sponsor or patients who are employees or relatives of the users
- Patients committed to an institution by virtue of an order issued either by the judicial or the administrative authorities

1. **SUPPLEMENTARY TABLES**

**ACOUSTIC QUALITY**

Table S1: Numeric rating of the acoustic quality of auscultations with stethoscope cover stratified by gender

| **Strata** | **Variable** | **Parameter** | **Result** | **p-value** |
| --- | --- | --- | --- | --- |
| **Gender** | Female |  |  | 0.0037 |
|  |  | N | 136 (25.5%) |  |
|  |  | Missings | 0 |  |
|  |  | Mean | 4.0 |  |
|  |  | SD | 0.7 |  |
|  |  | Median | 4.0 |  |
|  |  | Minimum | 2.0 |  |
|  |  | Maximum | 5.0 |  |
|  | Male |  |  |  |
|  |  | N | 396 (74.2%) |  |
|  |  | Missings | 2 |  |
|  |  | Mean | 4.2 |  |
|  |  | SD | 0.7 |  |
|  |  | Median | 4.0 |  |
|  |  | Minimum | 2.0 |  |
|  |  | Maximum | 5.0 |  |

Table S2: Acoustic quality of auscultations (numeric) with stethoscope cover stratified by BMI

| **Strata** | **Variable** | **Parameter** | **Result** | **p-value** |
| --- | --- | --- | --- | --- |
| **Body mass index** | Normal weight |  |  | <.0001 |
|  |  | N | 183 (34.3%) |  |
|  |  | Missings | 2 (0.4%) |  |
|  |  | Mean | 4.3 |  |
|  |  | SD | 0.6 |  |
|  |  | Median | 4.0 |  |
|  |  | Minimum | 2.0 |  |
|  |  | Maximum | 5.0 |  |
|  | overweight |  |  |  |
|  |  | N | 194 (36.3%) |  |
|  |  | Missings | 0 |  |
|  |  | Mean | 4.2 |  |
|  |  | SD | 0.8 |  |
|  |  | Median | 4.0 |  |
|  |  | Minimum | 2.0 |  |
|  |  | Maximum | 5.0 |  |
|  | Class I obesity |  |  |  |
|  |  | N | 123 (23.0%) |  |
|  |  | Missings | 0 |  |
|  |  | Mean | 4.0 |  |
|  |  | SD | 0.6 |  |
|  |  | Median | 4.0 |  |
|  |  | Minimum | 2.0 |  |
|  |  | Maximum | 5.0 |  |
|  | Class III obesity |  |  |  |
|  |  | N | 32 (6.0%) |  |
|  |  | Missings | 0 |  |
|  |  | Mean | 3.8 |  |
|  |  | SD | 0.8 |  |
|  |  | Median | 4.0 |  |
|  |  | Minimum | 2.0 |  |
|  |  | Maximum | 5.0 |  |

Table S3: Acoustic quality of auscultations (numeric) with stethoscope cover stratified by body site

| **Strata** | **Variable** | **Parameter** | **Result** | **p-value** |
| --- | --- | --- | --- | --- |
| **Body site** | Lung |  |  | <0.0001 |
|  |  | N | 191 (35.8%) |  |
|  |  | Missings | 2 (0.4%) |  |
|  |  | Mean | 4.3 |  |
|  |  | SD | 0.7 |  |
|  |  | Median | 4.0 |  |
|  |  | Minimum | 2.0 |  |
|  |  | Maximum | 5.0 |  |
|  | Abdomen |  |  |  |
|  |  | N | 177 (33.1%) |  |
|  |  | Missings | 0 |  |
|  |  | Mean | 4.2 |  |
|  |  | SD | 0.7 |  |
|  |  | Median | 4.0 |  |
|  |  | Minimum | 2.0 |  |
|  |  | Maximum | 5.0 |  |
|  | Heart |  |  |  |
|  |  | N | 154 (28.8%) |  |
|  |  | Missings | 0 |  |
|  |  | Mean | 4.1 |  |
|  |  | SD | 0.6 |  |
|  |  | Median | 4.0 |  |
|  |  | Minimum | 2.0 |  |
|  |  | Maximum | 5.0 |  |
|  | Other |  |  |  |
|  |  | N | 10 (1.9%) |  |
|  |  | Missings | 0 |  |
|  |  | Mean | 3.3 |  |
|  |  | SD | 0.7 |  |
|  |  | Median | 3.0 |  |
|  |  | Minimum | 2.0 |  |
|  |  | Maximum | 4.0 |  |
